# Increased Amoxicillin–Clavulanic Acid Resistance in *Escherichia coli* Blood Isolates, Spain

**DOI:** 10.3201/eid1408.071059

**Published:** 2008-08

**Authors:** Jesús Oteo, José Campos, Edurne Lázaro, Óscar Cuevas, Silvia García-Cobos, María Pérez-Vázquez, F. J. de Abajo, Spanish Members of EARSS

**Affiliations:** * Instituto de Salud Carlos III, Madrid, Spain; † Consejo Superior de Investigaciones Científicas, Madrid; ‡ Agencia Española del Medicamento y Productos Sanitarios, Madrid

**Keywords:** amoxicillin–clavulanic acid, sepsis, *Escherichia coli*, antibiotic resistance, dispatch

## Abstract

To determine the evolution and trends of amoxicillin–clavulanic acid resistance among *Escherichia coli* isolates in Spain, we tested 9,090 blood isolates from 42 Spanish hospitals and compared resistance with trends in outpatient consumption. These isolates were collected by Spanish hospitals that participated in the European Antimicrobial Resistance Surveillance System network from April 2003 through December 2006.

In addition to being an essential component of the gut flora, *Escherichia coli* is an etiologic agent for both hospital- and community-acquired infections in humans (*1**–**3*). As with other bacterial pathogens, this bacterium can develop resistance and multidrug resistance to several antimicrobial families; consequently, antimicrobial treatment of invasive *E. coli* infections can be challenging (*1*).

Amoxicillin–clavulanic acid (AMC) is one of the most consumed antimicrobial agents in many countries (*4**–**6*), principally for respiratory and urinary tract infections. However, little is known about its impact on antimicrobial drug resistance, particularly in *E. coli*. *E. coli* is one of the indicator organisms of the European Antimicrobial Resistance Surveillance System (EARSS) (*7*), an international network of surveillance systems that attempt to collect reliable and comparable antimicrobial resistance data on invasive pathogens (*1*).

## The Study

The 42 participating Spanish hospitals were selected according to EARSS criteria (*1**,**7*). The total catchment population was ≈9 million people, or ≈22.5% of the Spanish population. The first blood *E. coli* isolates obtained from each patient between 2003 and 2006 were included. Each laboratory identified the strains and tested their susceptibilities according to standard microbiologic procedures; all used commercial microdilution systems. Susceptibility data were interpreted according to Clinical Laboratory Standards Institute criteria (*8*). For epidemiologic purposes, intermediate susceptibility to AMC was considered as resistance. Multidrug resistance was defined as resistance to >3 of the following antimicrobial agents: ciprofloxacin, gentamicin, cotrimoxazole, and cefotaxime. To assess the comparability of susceptibility test results, an external quality assurance exercise (UK National External Quality Assessment Scheme) was performed yearly.

Hospital-acquired infections were defined as infections acquired at least 48 hours after hospital admission. Community-acquired infections were those in which *E. coli*–positive cultures were identified at or within 48 hours of hospital admission.

Outpatient consumption of penicillin/β-lactamase inhibitors (World Health Organization code J01CR02) for the period 2002–2006 was assessed from the Especialidades Consumo de Medicamentos database, which showed retail pharmacy sales of all medicines acquired with National Health System prescriptions and covered nearly 100% of the Spanish population (*5*). The information was tabulated, and the number of units was converted into defined daily doses (DDD) of active drug ingredients according to WHO methodology (*9*). The number of DDD per 1,000 inhabitants per day (DIDs) was calculated for each active drug ingredient.

Differences in the antimicrobial resistance prevalence between different groups were assessed by Fisher exact test. Association was determined by calculation of the odds ratio (OR) with 95% confidence intervals (CI). The significance of the antimicrobial resistance trends was calculated by χ^2^ test for trend. The null hypothesis was rejected for values of p<0.05. Statistical analyses were performed by using GraphPad Prism version 3.02 software (GraphPad Software, Inc., San Diego, CA, USA).

Participating hospitals reported data on 9,090 cases of *E. coli* bacteremia during the study period, corresponding to the same number of patients; 4,526 (49.8%) were male patients and 4,564 (50.2%) were female patients. A total of 1,531 cases (16.8%) were diagnosed in 2003; 2,526 (27.8%) in 2004; 2,438 (26.8%) in 2005; and 2,597 (28.6%) in 2006. Of the total number of isolates, 328 (3.6%) were obtained from children <14 years of age; 2,857 (31.4%) were obtained from patients >15 and <64 years of age; and 5,909 (65%) were obtained from patients >64 years of age. There were 3,384 (37.9%) isolates implicated in hospital-acquired infections and 5,540 (62.1%) in community-acquired infections; information was missing for 166 cases.

Of the 9,090 *E. coli* isolates tested, 1,136 (12.5%) were nonsusceptible to AMC, 5.1% were resistant, and 7.4% were intermediate. The prevalence of amoxicillin/clavulanic acid nonsusceptibility in relation to gender, age, infection origin, and resistance to other antibimicrobial drugs is detailed in the Table.

**Table:** Association of AMC nonsusceptibility of *Escherichia coli* from blood with gender, age, infection origin, and resistance to other antimicrobial agents, Spain, 2003–2006*

Variable	AMC nonsusceptibility, %	Odds ratio	95% Confidence interval	p value
Gender				
Male	13.6	1.23	1.08–1.39	0.001
Female	11.4			
Age				
<14 y	14.2	1.12	0.83–1.52	0.51
>14 y	12.8			
Infection origin				
Nosocomial	13.8	1.18	1.04–1.34	0.012
Community	12.0			
Antimicrobial susceptibility				
Ciprofloxacin susceptible	9.0	2.93	2.59–3.32	<0.0001
Ciprofloxacin resistant	22.5			
Gentamicin susceptible	11.5	2.64	2.22–3.14	<0.0001
Gentamicin resistant	25.6			
ESBL-negative	11.5	3.40	2.84–4.08	<0.0001
ESBL-positive	30.7			

*AMC, amoxicillin-clavulanic acid; ESBL, extended-spectrum β-lactamase.

Multidrug resistance was present in 198 (17.4%) of the nonsusceptible AMC isolates. The most prevalent phenotypes included multidrug resistance to ciprofloxacin, cotrimoxazole, and gentamicin, which was detected in 73 nonsusceptible AMC isolates (36.9% of multiresistant isolates and 6.4% of isolates overall), and resistance to ciprofloxacin, cotrimoxazole, and cefotaxime was detected in 55 isolates (27.8% of multiresistant isolates; 4.9% of isolates overall). Multidrug resistance was more prevalent in nosocomial (23.5%) than in community-acquired isolates (15.1%; OR 1.99, 95% CI 1.46–2.72; p<0.0001).

Among nonsusceptible AMC isolates, susceptibility to other antimicrobial drugs, including ciprofloxacin, gentamicin, cefotaxime, and cotrimoxazole, was more frequent in community- (28.7%) than in hospital-acquired isolates (13.3%; OR 1.68, 95% CI 1.27–2.22; p = 0.0003). This suggests that more therapeutic options were available for community-acquired isolates.

**Figure 1:**
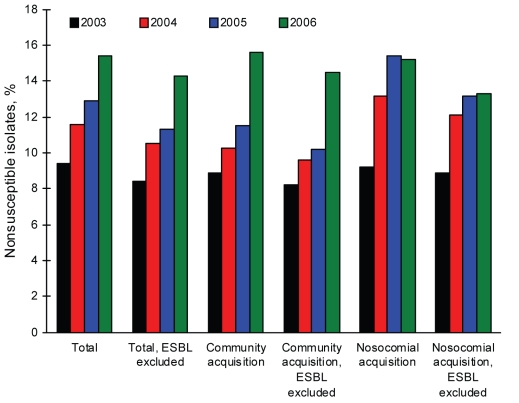
Evolution of amoxicillin-clavulanic acid nonsusceptibility of *Escherichia coli* from blood isolates, Spain, 2003–2006. ESBL, extended-spectrum β-lactamase.

The overall rate of invasive *E. coli* nonsusceptibility to AMC increased from 9.3% (2003) to 15.4% (2006) (χ^2^ test for trend 36.51; p<0.0001) (Figure 1); this increase was observed in 64.3% of the participant hospitals. This increase was also detected in both intermediate and resistant isolates, with annual distributions of 5.6% and 3.8%, respectively, in 2003; 6.8% and 4.8% in 2004; 7.5% and 5.4% in 2005; and 9.4% and 6% in 2006.

AMC nonsusceptibility according to age groups increased over the study period as follows: children <14 years of age (10.6% in 2003, 14.6% in 2004, 14.3% in 2005, and 16.3% in 2006); patients >15 and <64 years 9.6% in 2003, 11.2% in 2004, 11.7% in 2005, and 13.3% in 2006); patients >64 years (8.8% in 2002, 11.3% in 2004, 15.9% in 2005, and 16.3% in 2006). The prevalence of AMC nonsusceptibility in community-acquired infections increased from 8.9% (2003) to 15.6% (2006) (χ^2^ test for trend 29.43; p<0.0001). AMC nonsusceptibility in nosocomial infections increased from 9.2% (2003) to 15.2% (2006) (χ^2^ test for trend 11.94; p = 0.0006).

In the final 2 years of the study period (2005–2006), the proportion of AMC-nonsusceptible isolates increased from 12.9% to 15.9%. This increase was due to community-acquired *E. coli* isolates only; the nonsusceptible proportion varied from 11.5% (2005) to 15.6% (2006) (OR 1.42, 95% CI 1.16–1.74; p = 0.0009) in community-acquired isolates compared with 15.4% (2005) to 15.2% (2006) in hospital-acquired isolates (Figure 1). Community-acquired infection probably included healthcare-associated infections, a recently described epidemiologic category distinct from both community-acquired and nosocomial status.

In this study, the number of blood isolates of *E. coli* producing extended-spectrum β-lactamase (ESBL) was 614 (6.7%); 188 of them (30.6%) were nonsusceptible to AMC. When ESBL-producing *E. coli* isolates were excluded from analysis, AMC nonsusceptibility increased from 8.4% (2003) to 14.3% (2006) (χ^2^ test for trend 34.39; p<0.0001) in total isolates; from 8.2% (2003) to 14.5% (2006) (χ^2^ test or trend 25.23; p<0.0001) in community-acquired isolates; and from 8.9% (2003) to 13.3% (2006) (χ^2^ test for trend 6.35; p = 0.012) in hospital-acquired isolates (Figure 1). The proportion of isolates highly susceptible to AMC (MIC <4 mg/L) steadily decreased over the study period as follows: 70.2% (2003), 70% (2004), 64.8% (2005), and 57.4% (2006) (χ^2^ test for trend 99.36; p<0.0001).

**Figure 2:**
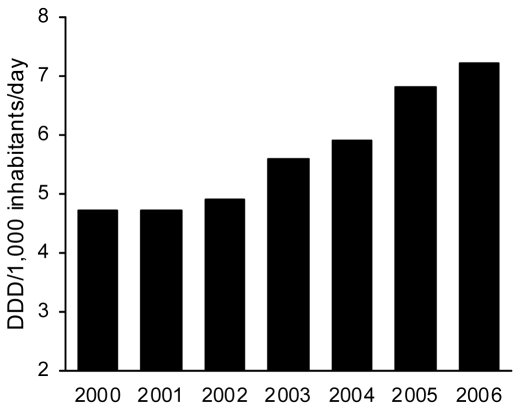
Evolution of consumption of outpatient penicillin/β-lactamase inhibitors (World Health Organization code J01CR02), Spain, 2000–2006. DDD, defined daily dose.

Community consumption of penicillin/β-lactamase inhibitors, predominantly AMC, increased 34.7% from 2000 to 2006 (Figure 2), whereas total antimicrobial drug consumption remained relatively constant (19.6 DIDs in 2000 compared with 19.1 DIDs in 2006). After AMC, the most used β-lactam antimicrobial agents in the community in Spain were amoxicillin, cefuroxime, and cefixime; their consumption did not vary or slightly decreased from 2002 through 2005 (*6*).

## Conclusions

The increased AMC resistance of *E. coli* isolates from blood observed in this study is of serious concern from clinical and epidemiologic standpoints because AMC is the first-choice antimicrobial treatment for many invasive *E. coli* infections*.* Increased AMC resistance coincided with growing AMC consumption at the community level. In urinary infections, previous treatment with AMC is a risk factor for the development of AMC resistance (*10*). AMC resistance mechanisms (β-lactamase overproduction, AmpC cephalosporinase hyperproduction, and inhibitor-resistant penicillinases) (*11*) might be favored by strong AMC consumption.
